# Providing a Clean Environment for Adolescents: Evaluation of the Tobacco Hazards Prevention Act in Taiwan

**DOI:** 10.3390/ijerph14060634

**Published:** 2017-06-13

**Authors:** Min-Li Chen, Li-Na Chou, Ya-Cheng Zheng

**Affiliations:** 1 Department of Respiratory Care and Graduate Institute of Nursing, Chang Gung University of Science and Technology, Chiayi Campus, Puzi City 61363, Chiayi, Taiwan; t73003@gmail.com; 2 Nursing Department, National Tainan Junior College of Nursing, Tainan 700, Taiwan; 3 Graduate Institute of Nursing, Chang Gung University of Science and Technology, Chiayi Campus and Chiayi Chang Gung Memorial Hospital, Puzi City 61363, Chiayi, Taiwan; s2006888@cgmh.org.tw

**Keywords:** tobacco hazards prevention, adolescent, clean environment, health promotion

## Abstract

Cigarette smoking not only damages the health of adolescents, but also contributes to air pollution. The Tobacco Hazards Prevention Act in Taiwan stipulates that cigarettes should not be sold to persons younger than 18 years. Therefore, schools should actively educate students and raise awareness of the Tobacco Hazards Prevention Act to reduce the level of damage to the health of adolescents and maintain good air quality. This study had two main goals: (1) to evaluate the stipulation that no person shall provide tobacco products to persons under the age of 18 and the effects of counseling strategies on store managers confirming customer ages before tobacco sale in southern Taiwan; and (2) to evaluate the situation of tobacco hazard prevention education conducted by school in southern Taiwan. A cross-sectional design was adopted for this study. Study I: The investigation involved an analysis of 234 retailers including convenience stores (n = 70), grocery stores (n = 83), and betel nut stalls (n = 81). The results indicated that among the 234 retailers, 171 (73.1%) of them routinely failed to confirm the buyers’ ages before allowing them to purchase tobacco. The number of retailers who exhibited failure to confirm customer ages before selling tobacco products had decreased from 171 (73.1%) to 59 (25.2%) and that of those who confirmed customer ages before selling tobacco products had increased from 63 (26.9%) to 175 (74.8%) after counseling strategies had been provided, thereby revealing statistical significance (χ^2^ = 11.26, *p* < 0.001). Study II: A total of 476 (89.1%) participants had received tobacco hazards prevention education and 58 (10.9%) had not. Among the various residential areas, the highest percentage of participants that did not received tobacco hazards prevention education located in the plane regions (8.4%). The government organizations should continue to adopt counseling strategies to reduce the rate of disobedience of the Tobacco Hazards Prevention Act by selling tobacco products to minors. Schools should pay close attention to tobacco hazard prevention education for junior high school students to ensure that such students are adequately educated about tobacco hazard prevention.

## 1. Introduction

The annual average level of particulate matter smaller than 2.5 μm (PM_2.5_) in southern Taiwan is consistently at least four times higher than the World Health Organization (WHO) Air Quality Guideline of 10 μg/m^3^ [1,2]. Studies indicates that a high concentration of PM_2.5_ is an environmental risk factor for health problems such as respiratory disease, cardiovascular disease, and cancer [3,4]. Cigarette smoking is a major source of air pollution that generates PM_2.5_. Studies have shown that the concentration of PM_2.5_ released through cigarette smoke is among the highest sources of air pollution [5,6]. Gerber used an automatic environmental tobacco smoke emitter to test commercial tobacco products and found that the mean concentration of PM_2.5_ released by 3R4F, P&S, and Virginia unfiltered cigarettes was within the range of 1525–1982 μg/m^3^ [7]. Air pollution, particularly the high concentration of PM_2.5_, is a critical problem in Taiwan; therefore, reducing cigarette smoke to maintain good air quality is an issue that requires attention.

Cigarette smoking is a growing problem among Taiwanese adolescents, and air pollution caused by smoking and secondhand smoke is a major environmental risk factor influencing adolescent health. Studies have shown that air pollution from smoking damages the health of not only adolescent smokers but also those exposed to secondhand smoke [8]. Study results have shown that active smoking and exposure to tobacco smoke are associated with an elevated risk of respiratory symptoms and cardiometabolic diseases among adolescents [9,10]. The findings of a prospective study on 4134 Taiwanese adolescents aged 12–14 years indicated that active smoking or exposure to environmental tobacco smoke are associated with an increased risk of chronic coughs and chronic bronchitis [11]. Reducing the adolescent smoking rate and secondhand smoke emissions to maintain air quality and protect adolescent health are imminent missions for public health care providers in Taiwan. 

Article 13 of the Tobacco Hazards Prevention Act in Taiwan stipulates that it is illegal to provide tobacco products to persons under the age of 18 years. However, according to the 2014 Global Youth Tobacco Survey, 45.7% of junior high school smokers had successfully purchased cigarettes and 52.8% were not prevented from purchasing cigarettes indirectly. Moreover, 75.8% of senior high school smokers had purchased cigarettes directly and 67.9% were not prevented from purchasing cigarettes indirectly [12]. The Consumers’ Foundation, Chinese Taipei conducted on-site inspections of 660 tobacco retailers in 22 counties and cities in 2013 to assess the sale of tobacco products to minors. They found that 52.6% of all convenience stores, supermarkets, betel nut stalls, and grocery stores illegally sold tobacco products to minors, among which the violation percentages were 38.8%, 59.3%, and 69.1% for convenience stores, grocery stores, and betel nut stalls, respectively [13], indicating that illegal tobacco product sales to minors in Taiwan is a serious problem. 

Article 20 of the Tobacco Hazards Prevention Act in Taiwan stipulates that government agencies and schools are required to actively provide education and engage in publicity campaigns against tobacco hazards. Studies have indicated that tobacco hazards prevention should be the highest educational priority for schools and communities [14]. Data from 2015 indicated that the smoking rate among junior high school students in Taiwan is 3.5% (boys: 4.9%; girls: 2.0%) [15]. The secondhand smoke exposure rate of junior high school students on campus is 7.5%. The primary source of exposure is classmates who smoke and the main secondary sources are extramural smokers and school administrative staff [15]. Although the smoking and secondhand smoke exposure rates have declined since 2013–2014 [15], the Tobacco Hazards Prevention Act in Taiwan stipulates that smoking is entirely prohibited at elementary, junior and senior high schools, making campuses smoke-free zones. Reducing the number of stores illegally selling tobacco products to minors, promoting tobacco hazards prevention, and reducing adolescent smoking and secondhand smoke exposure rates can all assist in the enhancement of clean air in schools and communities, and thus should be the top priorities of public health care providers. 

The literature shows that research on tobacco hazards prevention among junior high school students in Taiwan has focused on active smoking and secondhand smoke exposure [16], smoking-related behaviors and attitudes [17], and the effects of smoking on academic performance [18]. Few studies have analyzed the Tobacco Hazards Prevention Act in Taiwan and factors such as the illegal sale of tobacco products to minors and tobacco hazards prevention education in schools. Accordingly, the present study had two main goals: (1) to evaluate the stipulation that no person shall provide tobacco products to persons under the age of 18 and the effects of counseling strategies on store managers confirming customer ages before tobacco sale in southern Taiwan; and (2) to evaluate the situation of tobacco hazard prevention education conducted by school in southern Taiwan.

## 2. Materials and Methods

The present study was authorized to evaluate the stipulation of no person shall provide tobacco products to persons under the age of 18 by the Department of Health Bureau in southern Taiwan. For this study goal, the review process of the Institutional Review Board (IRB) could be waived because of legal orders.

Regarding the second study goal, this study was approved by the IRB of Chang Gung Hospital in southern Taiwan (approval number: 1024399B). Before conducting surveys, homeroom teachers described the study goals to the participants’ parents through parent–teacher contact books and obtained informed parental consent. Before completing the questionnaires, the homeroom teachers explained the study goals and protection of privacy to the participants. 

## 3. Design and Sampling

A cross-sectional design was adopted for this study:

Study I: The first study was conducted to investigate the illegal sale of tobacco products by tobacco retailers, including grocery stores, betel nut stalls, and convenience stores, located in mountainous, coastal, and plain regions in southern Taiwan. The number of samples of Tobacco retailers was based on the population of the county in this study. 25 tobacco retailers were selected in towns with a population of more than 50,000. Fifteen (15) tobacco retailers were selected in towns with a population of less than 50,000, and therefore 234 tobacco retailers were selected according the percentage of grocery stores, betel nut stalls, and convenience stores.

Study II: The second study was conducted to evaluate the situation of tobacco hazards education in schools by using a clustered random sample of students from 26 junior high schools in southern Taiwan (seventh, eighth, and ninth grade students). Two schools were selected each from mountainous regions, coastal regions, and plain regions. Subsequently, one class was selected from each grade of each school. Before the survey was conducted, two representatives from each school were selected to attend an introduction meeting regarding the study purpose and design. Upon returning to their schools, the attendees explained the study details to their homeroom teachers, who subsequently explained the study to the participants’ parents. While the participants were completing the questionnaires, the researchers were present to explain and clarify any confusion that may have arisen. This study was supported by all school principals and homeroom teachers from the selected schools. The effective response rate was 100%. 

## 4. Instruments

Study I: The survey testers were university students older than 18 years who wore uniforms to disguise themselves as high school students. They were assigned the task of purchasing tobacco products from tobacco retailers in various counties and cities in southern Taiwan. The testers assessed whether the retailers requested proof of age before selling them tobacco products. Tobacco retailers who sold tobacco products to people under 18 would receive counseling strategies including the following: (1) Multimedia teaching materials in the “Stop, Observe, and Listen” Manual [19] were supplied to these retailers. “Stop” refers to stopping the illegal sale of tobacco products to minors. “Observe” refers to observing whether the customer is a minor and requesting identification if in doubt. Finally, “listen” refers to retailers informing customers of the regulations and advising customers to abide by them. (2) Display posters warning against the illegal sale of tobacco products to minors were supplied to these retailers to remind them of the regulations prohibiting the sale of tobacco products to minors. The counseling strategies were provided by register nurses of health bureau. The effect of counseling strategies was evaluated after 3 months by the survey testers. 

Study II: A tobacco hazards prevention questionnaire was used for the second study. The questionnaire was formulated on the basis of a 2014 survey of junior high school student smoking behaviors conducted by the Health Promotion Administration, Ministry of Health and Welfare [12] and a review of the literature. The questionnaire consisted of two parts: (1) demographic characteristics including sex, age, residential area, smoking behavior, smoking habits of family members, secondhand smoke exposure based on the statement, “someone on campus has smoked in front of me within the past 7 days”[20], stores where the participants frequently purchases tobacco products, and received tobacco hazards prevention education; and (2) knowledge of tobacco hazards prevention assessed by 30 yes/no questions related to aspects such as no smoking arenas, penalty polices, and Article 13 of the Tobacco Hazards Prevention Act. One point was awarded for each correct answer; no points were awarded for incorrect answers. 

The questionnaire was evaluated by experts and a content validity index of 0.86 was determined. A total of 100 junior high school students were selected to pretest the questionnaire. The test–retest reliability in a 2-weeks interval was 0.82 based on the intraclass correlation coefficient. The Kuder–Richardson formula for reliability (KR20) based on questions related to the smoking behaviors of participants, smoking habits of their family members, and stores the participants frequently purchase tobacco products was 0.88. 

## 5. Statistical Analysis

SPSS Statistics 17.0 (SPSS Inc., Chicago, IL, USA) was used for the statistical analysis. Descriptive statistics (frequency distributions and percentages) were used to describe the participants’ demographic characteristics. The tobacco hazards prevention questionnaire results are expressed as percentages, means, and standard deviations. Pearson’s chi-squared test was employed to test for differences in the effect between pre-counseling strategies and post-counseling strategies supplied to retailers who disobeyed Article 13. One-way analysis of variance was conducted to test the relationships between the variables and cigarette smoking behaviors of the participants. Significant differences were subjected to Scheffe’s post hoc test.

## 6. Results

Table 1 shows details regarding the failure to confirm customer ages when selling tobacco products prior to the counseling strategies being supplied. The investigation involved an analysis of 234 retailers including convenience stores (n = 70), grocery stores (n = 83), and betel nut stalls (n = 81). The results indicated that among the 234 retailers, 171 (73.1%) of them routinely failed to confirm the buyers’ ages before allowing them to purchase tobacco. The results varied significantly between convenience stores, betel nut stalls, and grocery stores (χ^2^ = 5.12, *p* < 0.05), with 64 grocery stores having the highest rate of failure (27.2%) and 53 convenience stores having the lowest rate of failure (22.6%). 

Table 2 shows details regarding the failure to confirm customer ages when selling tobacco products after counseling strategies had been supplied. The findings of this study found that the number of retailers who exhibited failure to confirm customer ages before selling tobacco products had decreased from 171 (73.1%) to 59 (25.2%) and that of those who confirmed customer ages before selling tobacco products had increased from 63 (26.9%) to 175 (74.8%) after 3 months, thereby revealing statistical significance (χ^2^ = 11.26, *p* < 0.001).

Table 3 shows the participant demographics. This study selected 534 students aged 13–15 years from 26 junior high schools in southern Taiwan. The average participant age was 14.04 ± 1.7 years. Among the participants, 284 (53.2%) were boys and 250 (46.8%) were girls. Regarding residential area, 197 (36.9%) participants lived in plain regions, 180 (33.7%) lived in mountain regions, and 157 (29.4%) lived in the coastal regions. A total of 93 participants exhibited smoking-related behaviors, consisting of 65 who had tried a cigarette at least once (12.2%) and 28 who were daily cigarette smokers (5.2%). Among these, 82 participants (15.3%) were boys, 11 were girls (2.1%), 25 were aged 13 years (4.7%), 32 were aged 14 years (6.0%), and 36 were aged 15 years (6.7%). A total of 336 family members (62.9%) demonstrated smoking habits, among which the highest proportion was fathers with 128 (23.9%), while the second and third highest were grandparents 71 (13.3%) and relatives 67 (12.5%), respectively. Regarding secondhand smoke sources on campus, 149 (27.9%) individuals were extramural smokers and 92 (17.2%) were school administrators.

Table 4 shows details regarding the degree of understanding of tobacco hazards prevention. The lowest degree of understanding was observed for “there is a fine for disobeying tobacco hazards regulations”(38.80%), the second lowest was observed for “banning selling tobacco products to persons under the age of 18” (43.25%), and the highest was observed for “smoking shall be banned in public arenas” (61.70%).

Table 5 shows variations in knowledge of tobacco hazards prevention among the sex and age groups. The results show a significant difference between girls and boys in the degree of understanding of tobacco hazards prevention (*p* < 0.01). Girls showed a better understanding of tobacco hazards prevention than did boys. In addition, significant differences were observed for tobacco hazards prevention recognition among participants of different ages. Participants aged 14 years exhibited the highest degree of understanding of tobacco hazards prevention (*p* < 0.001).

Table 6 shows details of stores where tobacco products were frequently purchased. Most participants purchased tobacco products from grocery stores 46 (49.5%). The second highest proportion was for convenience stores 26 (27.9%) and the lowest was for betel nut stalls 21 (22.6%).

Table 7 shows details of participants received tobacco hazards prevention education. A total of 476 (89.2%) participants had received tobacco hazards prevention education and 58 (10.8%) had not. Among the various residential areas, the highest percentage of participants did not received tobacco hazards prevention education located in plain regions (8.4%). The results revealed a significant difference (χ^2^ = 45.13, *p* < 0.001).

## 7. Discussion

Almost all convenience stores, betel nut stalls, and grocery stores situated close to high schools in Taiwan display and sell tobacco products, making them convenient for students to purchase such products. Although the Tobacco Hazards Prevention Act explicitly stipulates that the parents, guardians or other people actually in charge of the care of persons under the age of 18 shall forbid said persons to smoke, stores tend to be driven by profit rather than protecting the physical and mental health of adolescents, and thus they are prepared to sell tobacco products to minors. The results of this study show that grocery stores exhibited the highest rate of selling tobacco products to minors, and the participants frequently purchased tobacco products from these stores, likely because the clerks at these stores did not routinely check consumer ages. In addition, the results demonstrate that the rate of stores selling tobacco products to minors was significantly reduced after stores had been supplied with counseling strategies. Heavy penalties are to be imposed on those who continue to disobey the act after counseling; such penalties are intended to prohibit this type of conduct and eliminate the sale of tobacco products to minors.

Among the junior high school students from southern Taiwan, the prevalence of cigarette smoking (17.4%; boys: 15.3%; girls: 2.1%), which included having tried a cigarette at least once and daily cigarette smoking, was equal to those of a sample of junior high school students with similar characteristics in Tainan City [21]. The results of that study demonstrated that the prevalence of daily cigarette smoking (5.2%) among junior high school students from southern Taiwan was higher than that observed by the national survey in Taiwan in 2015 (3.5%) [15] and lower than that of the same conditions reported among a sample of junior high school students with similar characteristics (6.6%) in Shanghai, China in 2013 [22]. Reductions in cigarette smoking exert the greatest positive impact on reducing the occurrence of health problems, such as respiratory disease, and cardiovascular disease, among adolescents [10,11]. Public health providers should develop tobacco control interventions tailored to supporting smoke-free environments by reducing cigarette smoking rates among adolescents.

The results of this study show that 336 (62.9%) family members of the participants exhibited smoking habits. Previous studies have indicated that smoking among fathers, mothers, siblings, and grandparents exert the strongest influence on the smoking behaviors of junior high school adolescents [23]. Moreover, studies have indicated that smoking when pregnant could increase the likelihood of the child developing a smoking habit as an adolescent [24]. In addition, classmates and school teachers exert fairly significant influences on adolescent smoking or quitting behaviors [25,26]. Parents and school teachers serve as examples of smoke prohibition and supervise adolescents while encouraging mutual support among classmates to prevent adolescent smoking, reduce smoking rates, and enhance the motivation to quit smoking. Therefore, the education of adolescent tobacco hazards prevention requires the participation of family members, classmates, and school teachers to reinforce its effectiveness [27,28].

Educational and governmental organizations could effectively reduce adolescent smoking rates and increase the motivation to quit through tobacco hazards prevention education [29]. The lowest degree of understanding was observed for “there is a fine for disobeying tobacco hazards regulations” and the second lowest was observed for “banning selling tobacco products to persons under the age of 18”. Governmental and educational organizations could use media strategies geared toward adolescents such as internet-based strategies, apps, flyers displayed on campus, and student clubs to spread awareness of the Tobacco Hazards Prevention Act, specifically focusing on “there is a fine for disobeying tobacco hazards regulations” and “banning selling tobacco products to persons under the age of 18”. Multimedia education with tobacco hazards prevention content designed based on adolescent culture and value could effectively facilitate the recognition of tobacco hazards prevention among adolescents to promote positive attitudes and behaviors [30]. 

In the present study, the female participants exhibited a stronger understanding of the Tobacco Hazards Prevention Act than did the male participants, which is consistent with the findings of Wu [21]. The participants aged 14 years exhibited a stronger understanding of the Tobacco Hazards Prevention Act than did those aged 13 and 15 years, possibly because Tobacco Hazards Prevention Act content is taught to eighth grade students, and thus the 14-year old participants had to actively collect relevant information for class assignments, thereby enhancing their awareness of tobacco hazards prevention. School-based tobacco prevention programs have effectively enhanced knowledge of the negative effects of cigarette smoking [31]. Providing smoking prevention programs may be critival in reducing smoking behaviors among adolescents. Moreover, tobacco hazards prevention education should be enhanced to prevent adolescent who have tried a cigarette at least once in developing smoking habits. Statistical data shows that smoking is commonly first practiced by junior high school students aged 13–15 years [15] and adolescent smoking behavior is positively correlated with continued smoking behavior in adulthood [32]. Therefore, tobacco hazards prevention should be taught in elementary schools to deepen students’ understanding of tobacco hazards and reinforce informed opinions toward smoking. 

The contents of the Tobacco Hazards Prevention Act are described in health education classes in Taiwan. The highest percentage of participants did not received tobacco hazards prevention education was observed at junior high schools located in plain regions, possibly because health education curriculums do not involve examinations for senior high school admission in Taiwan. Furthermore, some schools replace this class with other entrance examination classes. Therefore, the scenario of health education being overlooked could become worse in junior high schools in cities than in those in the countryside because schools in cities bear more pressure from the senior high school entrance examinations [33]. The Department of Education in each county and city should develop the counseling and supervisory strategies to ensure that schools conduct tobacco hazards prevention education, thereby not only effectively implementing Article 20: “Government agencies and schools shall actively engage in education and publicity campaigns against tobacco hazards”, but also ensuring that every junior high school student has access to information about tobacco hazards prevention.

In the present study, the primary source of secondhand smoke exposure was from extramural smokers on campus, which deviates from the statistical data of the Ministry of Health and Welfare at the Executive Yuan, which indicates that the primary source is classmates who smoke and the main secondary source is extramural smokers [15], possibly because “Friendly campus”, the policy of resource-sharing among schools and nearby communities, results in schools opening their campus spaces and facilities to community residents, thereby enabling extramural smokers on campus to generate secondhand smoke. To reduce the damage to air quality on campus and students’ health from secondhand smoke from extramural smokers, smoking ban slogans could be displayed on campus and tobacco hazards prevention issues could be broadcast on televisions to continually remind extramural smokers not to smoke on campus to allow students to enjoy a campus with clean air.

Public health providers play a vital role in addressing the impact of cigarette smoking on environmental change and tobacco hazards prevention by communicating why such environment change is a serious problem that could be hazardous to the adolescent population. Tobacco hazards prevention classes in elementary, junior high, and senior high schools in Taiwan emphasize only that tobacco hazards damage human health without mentioning that tobacco hazards are a major source of air pollution, which also influences human health. The effect of cigarette smoking on air quality should be incorporated into health education classes to enable students to understand that tobacco hazards prevention could protect not only their own health but also the environment, thereby enhancing their motivation to refuse to smoke or quit smoking. In addition to counseling strategies and fines being imposed on stores selling tobacco products to minors, governmental organizations should stipulate that store employees must participate in tobacco hazards prevention classes, thereby reinforcing their knowledge of environmental protection and enabling every individual to collaborate in protecting the environment. 

The results of this study illustrate that the lowest degree of understanding was observed for “there is a fine for disobeying tobacco hazards regulations” and the second lowest was observed for “banning selling tobacco products to people under the age of 18”. Such findings are the result of limited funds, labor, and materials for reinforcing tobacco hazards prevention education intervention programs, which could enable individuals to enhance their understanding of the Tobacco Hazards Prevention Act. Future studies could expand the study scope to different counties and cities by conducting longitudinal studies and increasing the usage of multimedia education programs, thereby enhancing the effectiveness of tobacco hazards prevention and rendering the research findings more representative, and thus enabling the findings to serve as a reference for promoting practical tasks related to tobacco hazards prevention.

## 8. Conclusions

The counseling strategies in this study involved using the “Stop, Observe, and Listen” Manual to remind stores selling tobacco products of points of attention to effectively increase the rate of confirming customer ages before making a tobacco sale from 26.9% to 74.8%. The Department of Health Promotion Administration could continue adopting such counseling strategies to reduce the rate of disobeying the Tobacco Hazards Prevention Act by selling tobacco products to minors. Schools should pay close attention to tobacco hazards prevention education for junior high school students to ensure that such students are adequately educated about tobacco hazards prevention. Health authorities worldwide play a critical role in increasing awareness of tobacco hazards prevention and should call upon the public to effectively assist the prohibition of disobeying restrictions on selling cigarettes to adolescents, thereby reducing adolescent smoking rates. Such efforts could not only reduce PM_2.5_ levels but also facilitate life quality maintenance through the reduction of medical expenses and air pollution due to the prevention of smoking. 

## Figures and Tables

**Table 1:** Failing to confirm customer ages when selling tobacco products prior to the counseling strategies being supplied (n = 234).

Retailers	Failing to Confirm Customers’ Ages before Tobacco Sale		X^2^
	Yes	No	
Convenience stores	53 (22.6%)	17 (7.3%)	5.12 *
Betel nut stalls	54 (23.2%)	27 (11.6%)	
Grocery shops	64 (27.2%)	19 (8.1%)	

* *p* < 0.05.

**Table 2:** Failing to confirm customer ages when selling tobacco products after counseling strategies had been supplied (n = 234).

Variables	before Counseling Strategies Had Been Supplied		after Counseling Strategies Had Been Supplied		χ^2^
Failing to confirm customers’ ages before tobacco sale	Convenience stores	53 (22.6%)	Convenience stores	10 (4.1%)	11.26 ***
	Betel nut stalls	54 (23.2%)	Betel nut stalls	26 (11.1%)	
	Grocery shops	64 (27.2%)	Grocery shops	23 (10.0%)	
	Total	171 (73.1%)	Total	59 (25.2%)	
Confirm customers’ ages before tobacco sale	Convenience stores	17 (7.3%)	Convenience stores	60 (25.6%)	
	Betel nut stalls	27 (11.6%)	Betel nut stalls	55 (23.5%)	
	Grocery shops	19 (8.1%)	Grocery shops	60 (25.7%)	
	Total	63 (26.9%)	Total	175 (74.8%)	

*** *p* < 0.001.

**Table 3:** Demographic data (n = 534).

Variables	n	%
Sex		
Male	284	53.2
Female	250	46.8
Age (14.04 ± 1.7)		
13 years old	159	30.0
14 years old	193	36.1
15 years old	182	33.9
Residential area		
Mountain regions	180	33.7
Coastal regions	157	29.4
Plain regions	197	36.9
Smoking		
Yes	93	17.4
Male	82	15.3
Female	11	2.1
(Had tried a cigarette at least once;	65	12.2
Daily cigarette smoker)	28	5.2
No	441	82.6
Smoking age		
13 years old	25	4.7
14 years old	32	6.0
15 years old	36	6.7
Family member smoking behavior		
No	196	36.7
Yes	336	62.9
Father	128	23.9
Mother	33	6.2
Grandparents	71	13.3
Siblings	37	6.9
Relatives	67	12.5
Missing value	2	0.4
Someone on campus has smoked in front of me within the past 7 days		
School administrators	92	17.2
Classmates	24	4.5
Extramural smokers	149	27.9
No one	342	64.0
Received tobacco hazards prevention education		
Yes	476	89.1
No	58	10.9

**Table 4:** Degree of understanding of tobacco hazards prevention (n = 534).

Tobacco Hazards Prevention	Question Numbers	Min	Max	Mean	SD	Skewness	Correct Answers (%)
1. Banning selling tobacco products to persons under the age of 18	4	0	4	1.73	1.30	0.11	43.25
2. There should be non-smoking slogans marked in public arenas	3	0	3	1.68	0.89	−0.44	56.00
3. Smoking ban could protect children, adolescents and pregnant ladies	4	0	4	2.25	1.04	−0.51	56.25
4. Smoking shall be banned in public arenas	10	0	10	6.17	2.55	−0.55	61.70
5. There is a fine for disobeying tobacco hazards regulations	5	0	5	1.69	1.47	0.34	38.80
6. People could seek assistance from public interest groups to quit smoking	4	0	4	1.94	0.98	−0.43	48.50
Total	30	0	30	19.09	7.45	−0.30	48.95

**Table 5:** Variations in knowledge of tobacco hazards prevention among the sex and age groups (n = 534).

Variables	N	Mean	SD	T/F Value	Scheffe’s Test
Sex				2.72 **	
Female	284	20.07	6.97		
Male	250	18.27	7.74		
Age				12.12 ***	
13 years old	159	17.49	7.05		14 years old > 13 years old
14 years old	193	21.17	7.40		14 years old > 15 years old
15 years old	182	18.40	7.41		

** *p* < 0.01; *** *p* < 0.001.

**Table 6:** Stores where tobacco products were frequently purchased (n = 93).

Stores	n	%
Convenience stores	26	27.9
Betel nut stalls	21	22.6
Grocery shops	46	49.5

**Table 7:** Participants received tobacco hazards prevention education (n = 534).

Residential Area	Yes (%)	No (%)	χ^2^
Mountain regions	173 (32.4)	7 (1.3)	45.13 ***
Coastal regions	151 (28.3)	6 (1.1)	
Plain regions	152 (28.5)	45 (8.4)	
Total	476 (89.2)	58 (10.8)	

*** *p* < 0.001.
